# Chitin and Chitosan as Direct Compression Excipients in Pharmaceutical Applications

**DOI:** 10.3390/md13031519

**Published:** 2015-03-19

**Authors:** Adnan A. Badwan, Iyad Rashid, Mahmoud M.H. Al Omari, Fouad H. Darras

**Affiliations:** Research and Innovation Center (RIC), The Jordanian Pharmaceutical Manufacturing Co., P.O. Box 94, Naor 11710, Jordan; E-Mails: irashid@jpm.com.jo (I.R.); momari@jpm.com.jo (M.M.H.A.); fdarras@jpm.com.jo (F.H.D.)

**Keywords:** chitin, chitosan, direct compression, co-processed excipients, solid formulation

## Abstract

Despite the numerous uses of chitin and chitosan as new functional materials of high potential in various fields, they are still behind several directly compressible excipients already dominating pharmaceutical applications. There are, however, new attempts to exploit chitin and chitosan in co-processing techniques that provide a product with potential to act as a direct compression (DC) excipient. This review outlines the compression properties of chitin and chitosan in the context of DC pharmaceutical applications.

## 1. Introduction

The exploitation of new techniques in pharmaceutical processing has given preferential emphasis to the use of DC over wet granulation approaches to tablet production. Apart from the simplicity of formulation and manufacture, the key advantages of DC include reduced capital, labor, and energy costs, and the avoidance of water of granulation for water-sensitive drugs [1].

There exist two aspects that are necessary for DC processing to stand as a real industrial operation. The first one is the properties and functionalities of the inactive pharmaceutical ingredients (*i.e.*, excipients). As a general rule, it is industrially desirable to minimize the number of additives through the use of multifunctional excipients, typically needed to act as a diluent, filler, binder, glidant, disintegrant, or lubricant. The second one is the complexity of the powder modification process for the excipient to be successful as a DC excipient. Some processes represent highly effective techniques for powder modification that suit DC applications, e.g., spray drying [2]. However, the level of complexity of these processes with regard to operation and unit control is high and they may impose high costs and investment of time. Consequently, it is industrially desirable to utilize, for example, roll compaction for powder consolidation as an effective approach for dry granulation [3].

On the other hand, the choice of suitable excipients for DC is a critical decision. In addition, knowledge and understanding of the behavior of these excipients are important to avoid potential problems during manufacturing [4]. However, few excipients can be directly compressed into tablets, owing to detrimental physical properties such as poor compactibility, flowability, and compressibility [5]. Therefore, there is a need to improve such properties by physical or chemical co-processing modifications [6].

### 1.1. Chitin and Chitosan Production

Preparation of chitin and its derivatives, such as chitosan, was fully described by Daraghmeh *et al.*, in addition to its physico–chemical properties and methods of analysis [7]. Chitins are usually isolated from the shells of marine crustaceans, which are highly abundant as a waste product from seafood processing. Crustacean shells contain 30%–40% proteins, 30%–50% calcium carbonate, and 20%–30% chitin in addition to lipidic pigments such as carotenoids (astaxanthin, astathin, canthaxanthin, lutein, and β-carotene). The proportion of each component varies with species and with season. Upon extraction, chitin is subjected to acid treatment to remove calcium carbonate, followed by alkaline treatment to remove proteins and a depigmentation step to remove the coloring agents, in particular astaxanthin [8,9].

Chitosan is prepared by hydrolysis of chitin using severe alkaline treatment. When thermal treatments of chitin under strong aqueous alkali are used, partial deacetylation takes place (degree of acetylation (DA) lower than 30%), transforming chitin into chitosan. The foregoing can be achieved with sodium or potassium hydroxides at a concentration of 30–50% *w*/*v* at high temperature (100 °C) [8,9].

The DA and molecular weight (MW) are important parameters to be examined because they affect the physical and chemical properties of chitin and chitosan [8,10]. The acetylated units prevail in chitin (DA typically > 90%), while chitosan has a typical DA of less than 35%.

Different techniques have been used to measure the DA including IR, near IR, and NMR (^1^H NMR, ^13^C NMR) spectroscopy, in addition to pyrolysis gas chromatography, gel permeation chromatography, zero- and first-order derivative UV spectrophotometry, thermogravimetric analysis, potentiometric titration, acid hydrolysis and HPLC, separation spectrometry methods, and, more recently, near-IR spectroscopy [8,11,12].

### 1.2. Chitin and Chitosan: Physical and Chemical Properties

**Figure 1:**
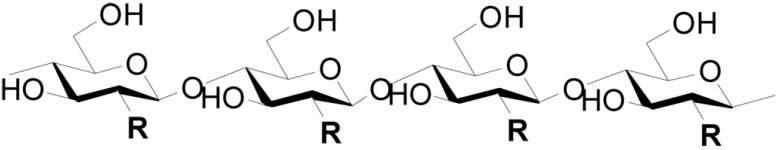
Chemical structures of cellulose (R = OH), chitin (R = NHCOCH_3_), and chitosan (R = NH_2_).

After cellulose, chitin is the second most abundant polysaccharide found in nature. Its chemical structure is similar to cellulose (Figure 1) which lacks the amine groups (thus chitin and chitosan are heteropolymers, while cellulose is a homopolymer) [8]. The presence of amino and hydroxyl groups in chitin and chitosan may offer opportunities to modify their chemical structures, subsequently improving their physical, chemical, and biological properties, including solubility [13].

As a result of extensive hydrogen bonding that promotes the semi-crystalline nature of chitin as well as its cohesive energy, chitin solubility is limited in all of the typical solvents, including aqueous and most organic solvents [13,14]. For example, chitin is considered soluble in hexafluoroacetone, hexafluoroisopropanol, and chloroalcohols when these solvents are mixed with mineral acids and dimethylacetamide in solutions containing 5% lithium chloride [14]. Strong polar protic solvents such as trichloroacetic acid and dichloroacetic acid in halogenated hydrocarbons were found to dissolve chitin. Furthermore, a mixture of calcium chloride and methanol acts as a good solvent combination for chitin [13].

On the other hand, chitosan is readily soluble in dilute acidic solutions below pH 6.0 (average pKa = 6.30) [13], which makes it a water-soluble cationic polyelectrolyte. As the pH increases above 6, chitosan loses its charge and becomes insoluble. The following salt forms of chitosan are considered soluble: acetate, formate, lactate, citrate, malate, glyoxylate, pyruvate, glycolate, and ascorbate [13]. However, the presence of glycerol 2-phosphate at a neutral pH of 7–7.1 offered an aqueous-based solution of chitosan at room temperature [15].

The presence of the reactive amino groups and the primary hydroxyl groups allow chitin and chitosan to be complexed or derivatized readily [16]. The chemical modifications may improve their physico-chemical properties, including solubility. The presence of water-soluble entities, hydrophilic moieties, and bulky and hydrocarbon groups extend their applications in various fields [13,14,15,17].

### 1.3. Applications of Chitin and Chitosan

The chelating ability of chitin and its derivatives facilitates their use as chromatographic and industrial adsorbents for the collection of industrial pollutants such as trace metals from aqueous and organic solutions, sea water, silver thiosulfate, and actinides [18,19,20]. In pharmacological applications, it was found that chitin activates peritoneal macrophages *in vivo*, whereas it suppresses the growth of tumor cells in mice, and stimulates nonspecific host resistance against *Escherichia coli* infection [21]. In addition, chitin accelerates wound healing [21,22]. Chitin derivatives are widely used to immobilize enzymes in the food industry and in other areas such as biosensors [21], and to immobilize antibodies in the presence of alginate [23]. They are also used as binders in the paper-making process [15]. Furthermore, chitin film and fiber are used in medical and pharmaceutical applications as wound-dressing material [22,24,25]. Recently, chitin has been involved in the conversion of biomass into a value-added, renewable nitrogen-containing furan derivative [26,27].

Chitosan has shown promise for use in different biomedical applications because of its high specific surface area and high porosity. For example, chitosan has been used in surgical sutures, dental implants, renewable artificial skin, bone rebuilding, contact lenses, controlled release of drugs for animals and humans, and as encapsulating material [15,28,29,30,31,32,33,34,35,36,37,38]. In addition, it has applications in agriculture, water and waste treatment, food and beverages, cosmetics and toiletries, and biopharmaceutical products [15,39].

## 2. Chitin and Chitosan for Direct Compression Processing

The suitability of chitosan for use in biomedical and pharmaceutical formulations is attributed to its inherent properties such as biodegradability, low toxicity, and good biocompatibility [40]. Among numerous pharmaceutical applications, it has been widely used as a vehicle for directly compressed tablets, a binder, a disintegrant, a granulating agent, and a carrier for sustained release preparations [17]. In solid dosage form preparations, chitosan has been used as a co-grinding diluent for the enhancement of the dissolution rate and bioavailability of water-insoluble drugs and as a penetration enhancer for peptide drugs [40].

Chitin and chitosan have been widely acknowledged as effective tablet disintegrants due to their high water absorption capacity. Such functionality was observed in tablets at chitin/chitosan concentrations below 70% [41]. Nonetheless, within the industrial framework, chitosan is not a common pharmaceutical additive in large-scale formulations. This is due to the fact that chitosan lacks good flow properties and compressibility [42]. Previous studies showed their limited use as fillers in directly compressed tablets [43,44], mainly due to their apparent low density, poor flow, and inadequate compressibility, resulting in tablets with very low mechanical strength. Even granulation or the inclusion of dry binders seemed to fail to induce the required mechanical strength.

For chitin, early attempts at its utilization as a pharmaceutical excipient were thoroughly investigated by Sawayanagi *et al.* [45]. They used physical mixing to produce binary mixtures of chitin with microcrystalline cellulose (Avicel PH 101^®^), lactose, or potato starch. They concluded that the flowability and tablet hardness when using the combined powders were greater than that when using microcrystalline cellulose, lactose, or potato starch as an individual excipient. The improvement of powder flow by the addition of chitin to microcrystalline cellulose (Avicel PH 102^®^) and co-processed lactose composed of a spray-dried mixture of 75% α-lactose monohydrate and 25% cellulose powder (Cellactose^®^) was recently confirmed by Mir *et al.* [46]. The bulk and tapped densities and the powder flow measured by the Carr Index (CI) and the angle of repose were all seen to increase upon the addition of chitin. The authors further pointed out that the highest CI was achieved with chitin alone, with a CI of 13.1% and a flow rate of 5.48 g/cm^2^ s. However, the inclusion of chitin in Avicel PH 102^®^ and Cellactose^®^ mixtures lubricated with magnesium stearate at a concentration of 0.5% *w*/*w* resulted in tablets with impaired mechanical properties. These results contradict the early findings of Sawayanagi *et al.* [45], probably due to the method of compression they were using. In the later work, a hydraulic tablet press with a compression duration time of 30 s was used for tablet compression whereas in the former work [46] a single station tableting machine was used with a dwell time generally estimated to be less than 0.1 s [47].

In studies carried out by Aucamp [43], Buys [48], and de Kock [44], it could be seen that chitosan could not be compressed into tablets on an eccentric tablet press. Aucamp came to the conclusion that even if combining chitosan with fillers such as Avicel PH 200^®^ or Prosolv^®^ SMC 90 (co-processed microcrystalline cellulose and colloidal silicon dioxide) the tablet strength was still weak. The combination of chitosan with the filler in the ratio 70:30 gave the best results. The conclusion was that the filler improved the flowability of the powder blend, resulting in better die filling and an increase in the tablet strength. The foregoing was correlated to the high surface area available for particle bonding resulting in decreased interparticular spaces (voids) [43]. Buys and de Kock compressed chitosan into mini-tablets. De Kock could not compress chitosan powder into tablets with desirable tablet strength; however, with the combination of binders and fillers chitosan mini-tablets could be obtained. Buys found that chitosan could only be compressed at high compression forces. It would be difficult to obtain these high compression forces needed to compress the powder when using an eccentric tablet press. The force exerted on the powder was achieved by adjusting the distance between the upper and lower punches. The problem of obtaining these higher compression forces was solved when a sufficient amount of chitosan powder filled the die before compressing the powder. These results concluded that although more chitosan powder could be filled into the die, the tablet weight was still relatively small.

**Figure 2:**
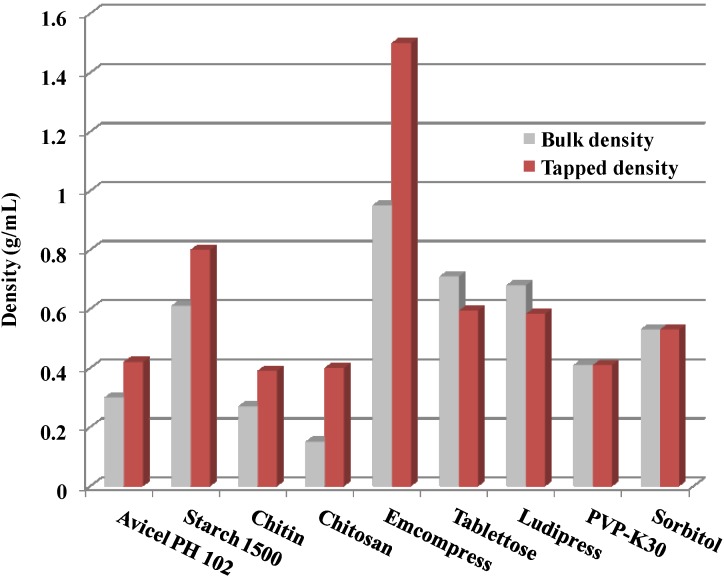
Bulk and tapped densities of chitin and chitosan in comparison with other common filler-binder excipients. Data were obtained from Mir *et al.* [46], Rojas *et al.* [52], and Sonnekus [53].

As a rule of thumb, bulk density is an indicator of a powder’s ability to undergo compression and compaction. Filler-binders or diluents generally provide the bulk of the tablet and are also responsible for flow and compaction properties. The bulk and tapped density of chitin and chitosan rank lowest when compared to other common filler binders and diluents (Figure 2) [9,46]. Such low density is attributed to their high particulate irregularities, which bring about a highly porous structure and a high specific surface area. In this regard, chitosan presents the highest specific surface area and pore volume among some common excipients presented in Table 1. Chitosan structure typically has significant internal void space in the form of pores and channels [49]. Moreover, the fibrous nature of chitin with irregular and some threaded surface will give rise to randomly sized but large voids (spaces between particles) between chitin/chitosan particles [50,51]. This can be justified by the big difference between bulk (0.15 g/mL) and tapped (0.40 g/mL) densities of chitosan powder (Figure 2), unlike the bulk (0.33 g/mL) and tapped (0.41 g/mL) densities of the almost spherically shaped particles of polyvinyl pyrrolidine (PVP K30). Similar conclusions can apply to microcrystalline cellulose due to its fibrous irregular particle shape.

**Table 1:** Specific surface area and pore volume of chitosan and some common excipients.

Excipient	Specific Surface Area (m^2^/g)	Pore Volume (cm^3^/g)	References
Lactose H_2_O	0.26	0.090	[54]
Microcrystalline cellulose	0.42	1.67	[55,56]
Maize starch	0.58	0.0012	[57]
Synthesized CaHPO_4_	3.31	0.0065	[58]
Chitosan	330	15	[59]

Microcrystalline cellulose: Avicel^®^ PH 101.

### 2.2. Powder Flow

The flow properties of the powder mixture are important to ensure mass uniformity of a tablet and thus prevent unacceptable variation in thickness, disintegration time, and strength [60]. On the basis of bulk and tapped densities, the powder flowability can be measured. For example, the CI is an indication of the flowability, and indirectly the compressibility, of the powder, as calculated according to Equation (1):

(1) $$Carr^{\prime }s\;Index\;\left(CI\right)=Compressibility\;\left(\%\right)=\frac{Tapped\;densit-Bulk\;density}{Tapped\;density}\times 100\%$$

**Figure 3:**
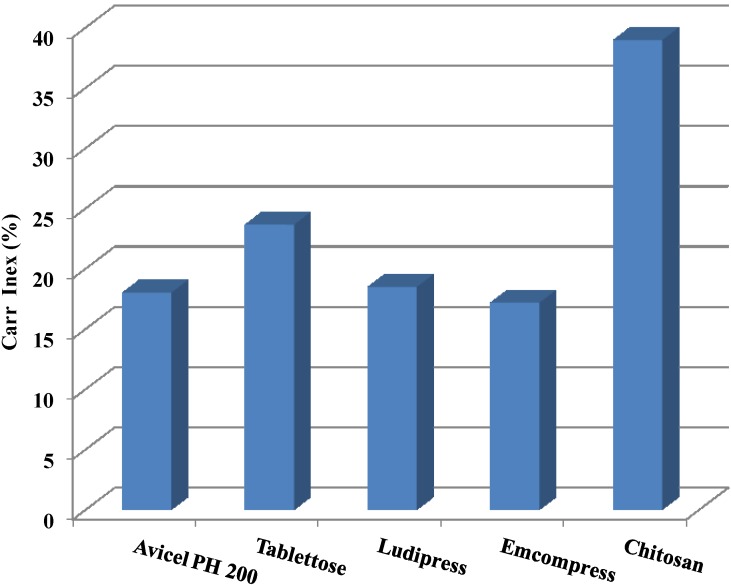
Carr Index (CI) of chitosan and some common direct compression excipients. Data were obtained from García Mir *et al.* [46], Rojas *et al.* [52], and Sonnekus [53].

CI values of 5–10, 12–16, 18–21, and 23–28 indicate excellent, good, fair, and poor flow properties of the material, respectively [61]. Therefore, it is clear that chitosan is not a free-flowing powder, and problems with filling a small die in a tablet press can be expected. Its compressibility is reported to be poor when compared to that of common DC excipients (Figure 3).

Generally, studies indicate that the flowability of powders containing spherical particles is much better than that of those containing elongated or irregular particles [62]. In this context, it was proposed that elongated or irregular particles obstruct powder flow and reduce flowability, as they tend to mechanically interlock or entangle with each other [63]. Thus the CI of chitosan was expected to be high because of its fibrous structure, which facilitates entanglements between particles, in addition to its cohesive nature resulting in adverse mechanical interlocking of powders with irregular shapes; consequently, poor flow properties are displayed [64].

The flowability of chitosan is affected by its moisture content and distribution at the time it is expected to flow. High moisture content negatively affects the flow of chitosan [65]. An increase in the relative humidity from 11% to 75% results in an increase in the CI from 30.7% to 36.1%. Moreover, chitosan’s flowability is affected by its particle size. The bigger the particle size, the better the flowability of chitosan. The smallest fraction (<90 μm) has a very poor flowability with a composite CI of 40.7%, while the fraction with a particle size of more than 212 μm has a CI of 29.7% [66].

### 2.3. Tensile Strength

**Figure 4:**
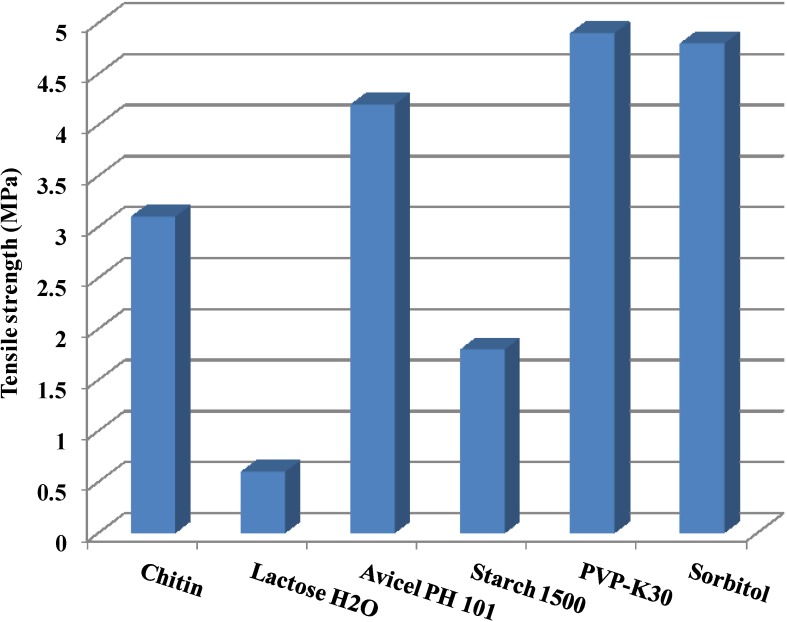
Tensile strength of chitin and some common direct compression excipients. Data were obtained from Rojas *et al.* [52,69].

The mechanical strength of tablets produced upon compression is the most essential requirement of DC excipients. With respect to chitin, their tablets manifest acceptable mechanical properties (Figure 4). This can be attributed to the irregular particle shape, the external surface area, and the rough surface texture of chitin; all contribute to the high surface bonding between the particles [67]. The tensile strength of chitosan tablets can be correlated with the plastic deformation nature of chitosan or with its volume reduction on compression. The volume reduction is associated with the elimination of pores. Generally, reducing compact pores results in a higher tensile strength of the tablets [68].

#### 2.3.1. Effect of Moisture Content

Moisture content plays a significant role in the consolidation and compaction of pharmaceutical powders [70,71,72,73,74]. The foregoing is attributed to the beneficial plasticizing effect of water on the polymer matrix or to the detrimental formation of stable hydrogen bonding bridges, resulting in an anti-plasticizing effect. [75]. These changes are a result of the combined effect of moisture on the inter-particle and intermolecular forces [72]. Accordingly, chemical or physical association between water and a polymer takes place, leading to a change in the behavior of water and of the polymer [76,77]. However, the extent of such association can alter the moisture action from that of a good tablet binder to a factor responsible for the weak tensile strength of tablets [73].

For chitosan, the tensile strength of the tablets compressed with dried chitosan powder were less at each of the compression forces used, when compared to that of tablets compressed with powder that was stored under atmospheric conditions containing 9.39% and 11.86% moisture [66]. In an attempt to elucidate the moisture-tensile strength effect, García Mir *et al.* attributed the effect, generally, to the ability of chitosan to hold a relatively large amount of water in its internal structure [78], and specifically to externally adsorbed water, which allows for the formation of hydrogen bonding between particles, thus preventing elastic recovery and/or increasing the interparticulate van der Waal’s forces. Consequently, the effects of surface micro-irregularities and inter-particle separation become reduced. On the other hand, excessive moisture provokes a disruption of the binding force between particles, resulting in a decrease in the tensile strength of tablets due to high absorbed water within the particle or condensed water in the surface [72]. Chitin, as a disintegrant, acts to enhance porosity and provide these pathways into the tablet. Liquid is drawn up or “wicked” into these pathways, replacing the air between the tablet particles, and thus weakening the intermolecular bonds [79].

#### 2.3.2. Effect of Degree of Deacetylation

The contribution of the degree of deacetylation (DDA) on the tensile strength has been previously studied by Gupta and Kumar. They pointed out that the hardness of chitin tablets was greater than that of chitosan tablets [80]. They attributed the difference in hardness to the structural rigidity of chitin due to its acetylamino groups and to the higher degree of polymerization typically found in chitin. The degree of polymerization of chitin would be reduced during the deacetylation of chitin with a strong alkali to prepare chitosan. The results of Rojas *et al.* confirm the aforementioned finding [52]. They pointed out that chitosan of low DDA has better mechanical properties compared to chitosan of a higher DDA. They contributed this phenomenon to the fact that chitin’s rigid structure is attributed to intra-sheet hydrogen bonds dominated by strong C–O**^…^**N–H hydrogen bonds, and to strong inter-molecular hydrogen bonds dominated by the amide groups, which undergo dramatic change into a less rigid orientation. The foregoing is a result when chitin is subjected to high NaOH concentrations and high reaction temperatures. Such hard conditions lead to a chitin with a low DA, low yield, and high crystallinity, which, in turn, forms weak compacts and has rapid disintegration times [52].

#### 2.3.3. Effect of Molecular Weight

The total length of the chitosan polymer is an important characteristic of the molecule. Hence, the MW is a key feature for its functional properties [81]. The first investigation of the effect of chitosan’s MW on tensile strength was carried out using chitosan films [82]. An increase in the MW of chitosan increased the tensile strength of the membrane because of intra- and inter-molecular hydrogen bonding [83].

**Figure 5:**
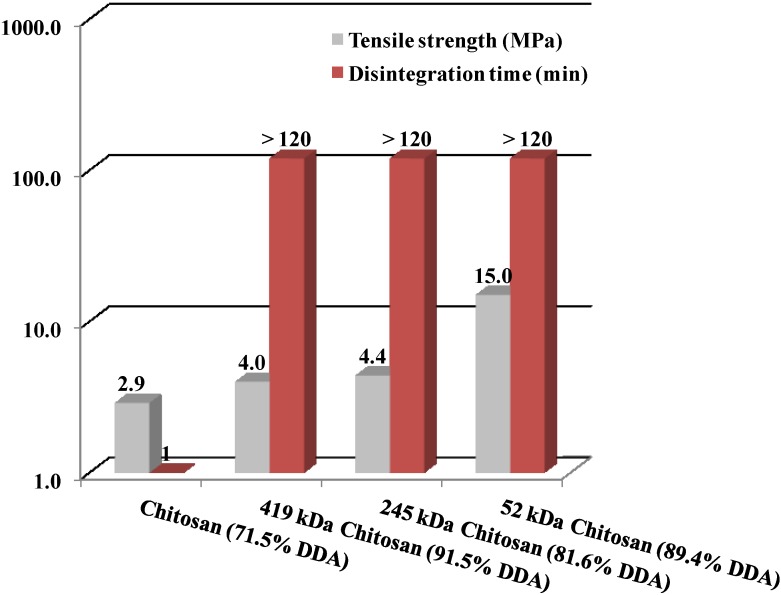
Tensile strength (MPa) and disintegration time (min) of chitin and chitosan with different molecular weights. Data were obtained from Rege *et al.* [42].

Rege *et al.* investigated tablet properties when using chitosan samples of different MW [42]. They demonstrated that a lower MW provides a higher tensile strength to a chitosan tablet (Figure 5). Consequently, low MW chitosans can be used to modify drug release to a greater extent than can high MW chitosana.

**Figure 6:**
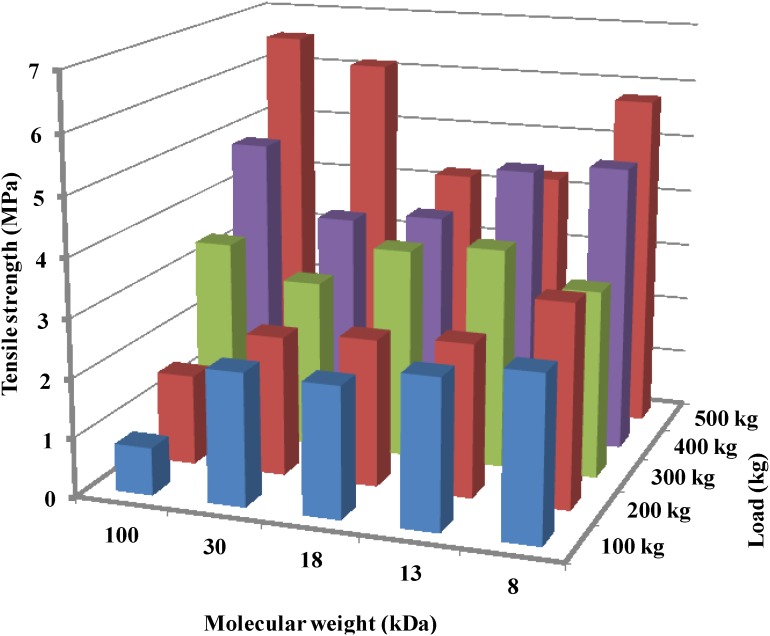
Effect of compression pressure on the tensile strength of tablets using chitosan samples of different molecular weights. Data were obtained from Rashid *et al.* [6].

Rashid *et al.* confirmed the aforementioned tensile strength dependency on MW; however, they used chitosan samples of much lower MW and lower compression pressures [6]. In their results, the highest MW chitosan (100 kDa) showed the highest compact tensile strength (Figure 6) at compression loads > 300 kg. They correlated such findings to the plastic deformation of chitosan, which was highest at 100 kDa, as indicated by its lowest yield pressure. Plastically deforming materials expose new surfaces upon compression and which are ready to provide further binding of the particles. On the other hand, the lowest MW chitosan samples (8 kDa) exhibited less plastic behavior or more brittle-fracture upon compression, in addition to lower elastic recovery. Such new fragments give rise to more binding surfaces and thereby high tensile strength. Therefore, Figure 6 illustrates the high tensile strength of chitosan when it is of a highly plastically deforming or a high brittle-fracture nature upon compression. Chitosans of MW between 100 and 8 kDa exhibit tensile strength values that are almost between that of the highest and lowest MW.

#### 2.3.4. Effect of Lubricant

**Figure 7:**
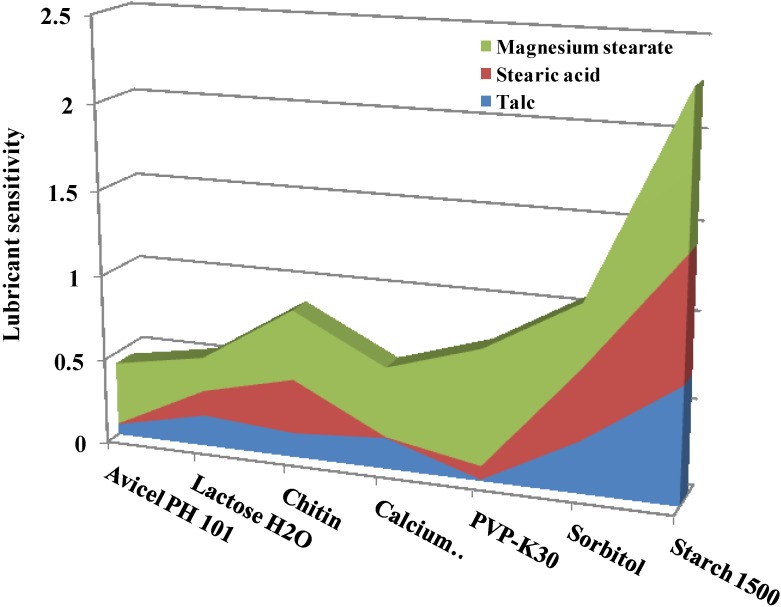
Lubricant sensitivity of chitin and common excipients in the presence of different lubricant types. Data were obtained from Rojas *et al.* [52,69].

In general, hydrophobic lubricants such as magnesium stearate have a negative effect on the crushing strength of plastically deforming constituents of tablets (e.g., microcrystalline cellulose) than on brittle ones (e.g., CaHPO_4_) [84]. As brittle materials are more likely to fracture and fragment during compaction, more fresh surfaces, not covered by lubricant particles, are generated. These new surface areas tend to bond together and thus the effect of lubricants is minimized. On the other hand, film formation by lubricants on plastically deforming particles weakens the bonding of the granules as there are fewer fresh surfaces formed during compaction. In general, hydrophobic lubricants provide efficient coating to the surface of particles and prevent the formation of hard compacts. This effect was more pronounced in highly plastic materials that had a smooth surface, such as pregelatinized starch. For chitin, despite its highly porous structure and its ability to undergo plastic deformation upon compression, its particle surface is highly irregular. Therefore, chitin’s external surface area dominates over other interparticulate bonding mechanisms upon compaction [67,85]. After Rojas *et al.* summarized common plastic and brittle excipient sensitivity to 1% lubricant [52], they concluded that because chitin was the material least sensitive to lubrication, it had better compactibility than pregelatinized starch, calcium diphosphate, and lactose monohydrate when lubricated (Figure 7).

The amount of magnesium stearate appeared to greatly affect the mechanical strength of chitin-containing tablets [46]. Tablets with a magnesium stearate concentration of 0.1% *w*/*w* exhibited a clearly higher crushing strength and lower friability than those compressed with a magnesium stearate concentration of 0.5% *w*/*w*. Picker-Freyer and Brink correlated the lubricant effect on tensile strength to the disruption of the microstructure of chitin [86]. Mir *et al.* attributed this effect to the reduction of interparticle bonding associated with chitin or a result of physical and chemical interaction between chitin and magnesium stearate [46]. Rashid *et al.* have shown that, upon mixing chitin with magnesium stearate, the specific surface area is considerably decreased while the particle size distribution remains unchanged [84]. On mixing with lubricant, microcrystalline cellulose undergoes a reduction in specific surface area followed by an increase in the particle size distribution, which has been correlated to agglomeration. Therefore, chitin exhibits high surface coverage by the lubricant, whereas microcrystalline cellulose exhibits particle agglomeration [84].

### 2.4. Compressibility of Chitin and Chitosan

Previous studies by Aucamp reveal poor compression characteristics for the pure chitosan raw material on an eccentric tablet press [43]. The unsuccessful attempt to produce tablets from pure chitosan with an average particle size of 215.6 μm was attributed to its poor compressibility and flow properties. From the results of a study by Buys [48], it was concluded that the poor compressibility of chitosan, resulting in tablets with low crushing strength and relative high friability, was due to the high porosity of chitosan. Therefore, the mass of chitosan filling the die was relatively low and, during compression, the tablet press could not sufficiently accomplish the necessary volume reduction of the material even at the highest compression setting.

Another drawback to the compression properties for chitosan is the relatively thin compacts produced to achieve an acceptable mechanical tablet strength and friability. Perioli *et al.* prepared tablets made from chitosan and PVP K90 or Polycarbophil, NOVEON^®^ AA-1 (PCP AA-1) and noted that the tablets’ width increased as the synthetic polymer content rose (PVP K90 > PCP AA-1), while it was inversely proportional to the amount of chitosan [87].

#### 2.4.1. Heckel Analysis

Compression properties determine the ability of powders to be compressed into tablets. These properties impart the tablet shape, porosity, and hardness, as well as the powder die-filling extent whereby the tablet weight is established. A number of mathematical models are available to analyze powder compression properties. Heckel analysis still remains the most powerful model in describing powder compressibility.

The Heckel equation (Equation (2)) is used for powder densification, where it describes the change of powder porosity as a function of the applied pressure [88]:

(2) $$\text{ln}\frac{1}{1-D}=kP+A$$

where *D* is the density of a powder compact at pressure *P*, and *k* is the slope of the data when presented as in Equation (1). The value of *k* is a measure of the plasticity of a compacted material. The inverse of the slope is the yield pressure of the materials (*P_y_*) [89], which describes the tendency of the material to deform either by plastic deformation or by brittle fracture. *P_y_* is inversely related to the ability of the material to deform plastically under pressure.

*A* is a constant related to the die filling and particle rearrangement before deformation and bonding of the discrete particles.

Two main parameters, *D_A_*, and *D_B_*, derived using the Heckel equation are helpful to assess the densification process. The relative density, *D_A_*, represents the total degree of densification, and the relative density, *D_B_*, describes particle rearrangement during the initial stages of compression. The two parameters are calculated from Equations (3) and (4):

(3) $$D_{A}=1-e^{-A}$$

(4) $$D_{B}=D_{A}-D_{o}$$

where *D*_0_ represents the relative density of powder at the point when the applied pressure is equal to zero.

When Heckel analysis is carried out for chitin in comparison with other common plastic and brittle excipients, the Heckel parameters can be summarized in Table 2. The yield pressure, which is a measure of the ductility of the materials, demonstrates that the plastic deformation of chitin is lower than that of PVP K30, which represented the highest plastically deforming excipient, followed by sorbitol, pregelatinized starch (Starch 1500), and Avicel PH^®^ 101. Calcium diphosphate as a brittle excipient presented the highest *P_y_* value or the lowest ductility, followed by lactose monohydrate [52].

**Table 2:** The Heckel parameters for chitin and different common DC excipients. Data were extracted from Rojas *et al.* [52,69].

Parameter	Calcium Diphosphate	Chitin	Lactose H_2_O	Avicel PH 101	Starch 1500	PVP K30	Sorbitol
*P_Y_*	250.1	122	150	62.5	75.1	35.7	48.4
*D*_0_	0.36	0.12	0.38	0.23	0.33	0.27	0.39
*D_A_*	0.49	0.52	0.69	0.44	0.48	0.72	0.79
*D_B_*	0.13	0.31	0.31	0.21	0.15	0.46	0.4

Starch 1500: pregelatinized starch, Avicel PH^®^ 101: microcrystalline cellulose.

**Figure 8:**
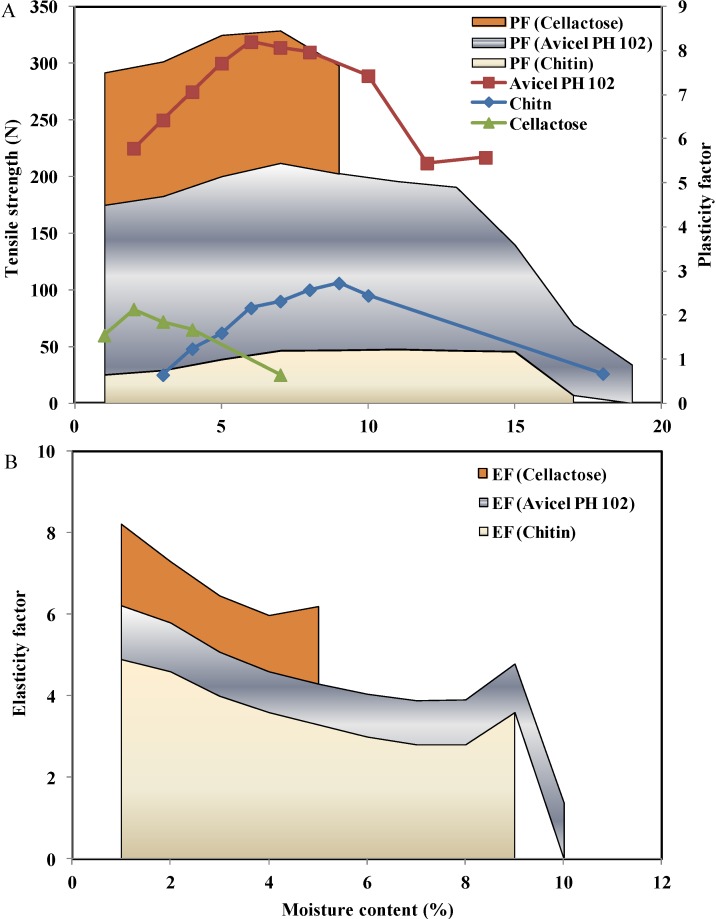
(**A**) Tensile strength and plasticity factor (PF) and (**B**) elasticity factor (EF) for chitin, microcrystalline cellulose (Avicel^®^ PH 102), and co-processed spray dried lactose (Cellactose^®^) as a function of moisture content. Data were obtained from Khan and Pilpel [71], and García Mir *et al.* [78].

Moreover, *D_A_*, *D_B_*, and *D*_0_, which represent the total degree of densification, the phase of rearrangement of particles during the initial stages of compression, and the relative density of powder when the applied pressure is equal to zero, are further presented in Figure 8. Sorbitol had the largest densification during die filling (*D*_0_); Avicel^®^ PH 101, along with chitin, had the lowest values. The low densification during die filling resulted in the thin compacts produced using chitosan-PVP K90 with high chitosan content [87]. On the other hand, PVP K30 and sorbitol presented the largest total particle densification (*D_A_*) and the largest rearrangement (*D_B_*) upon densification. Conversely, pregelatinized starch and calcium diphosphate presented the lowest rearrangement at initial compression pressures (*D_B_*). This is attributed to their initial large bulk densities and low porosity, causing small volume reduction at low pressures [52].

The low bulk density and low densification upon die filling of chitin or chitosan powders encouraged Buys to use a double filling cycle on a modified tablet press for the processing of chitosan in mini-tablets [48]. This has resulted in promising compression profiles for the material when the powder volume in the die cavity was increased through a double filling cycle. It was therefore postulated that if enough powder could be filled into the die cavity of a tablet press to increase the packing density of the material, then efficient particle bonding during compression should be able to produce tablets of acceptable mechanical strength.

#### 2.4.2. Kawakita Analysis

The compression behavior of the powder can also be evaluated using Kawakita analysis (Equation (5)). This model is dependent on the bulk density of the powder, unlike the true density, on which the Heckel model is dependent. The Kawakita equation is used to study powder compression via the degree of volume reduction:

(5) $$\frac{P}{C}=\frac{P}{a}+\frac{1}{ab}$$

where *C* is the degree of volume reduction of the powder column under the applied pressure *P*. The constant *a* is the minimum porosity of the material before compression. The constant *b* relates to the amount of plasticity of the material. The reciprocal of *b* (1/*b*), known as *P_k_*, defines the pressure required to reduce the powder bed by 50% [90,91]. It is worth noting that *P_k_* values are an inverse measure of the amount of plastic deformation occurring during compression [92].

#### 2.4.3. The Gurnham Equation

The Gurnham equation can be used to study the compression process in pharmaceutical powders [93]. In this model, any increase in pressure, expressed as a fractional increase over the existing pressure, is expected to result in a proportional increase in the apparent density of powder mass. According to Gurnham’s equation:

(6) $$\text{ε }\left(\%\right)=-cln{\text{σ}}_{c}+d$$

where *c* (the negative of the slope obtained by plotting ε (%) *vs.* *ln*σ*_c_* and *d* (the y-intercept) are constants. The constant *c* provides a good representation of material compressibility. Higher *c* values indicate better compressibility.

#### 2.4.4. Plasticity/Elasticity Factors

Upon compression, the plasticity and elasticity of materials can be measured using the force-distance curve near the maximum force by a method described by Antikainen and Yliruusi [94]. The plasticity factor (PF) determines the extent of plastic deformation at a certain compression force, as described in Equation (7):

(7) $$PF=\left(\;\frac{W_{1}}{W_{1}+W_{2}}\right)\times 100\%$$

where *W*_1_ and *W*_2_ are calculated from the force–displacement curve [94]. The elasticity factor (EF) can be calculated using Equation (8):

(8) $$EF=\left(\;\frac{S_{max}-S_{od}}{S_{max}-S_{o}}\right)\times 100$$

where *S_max_* is the maximum upper punch displacement, *S*_0_ is the displacement of the upper punch at first detection of force, and *S_od_* is the displacement of the upper punch in the decompression phase [94].

Without the need for double die filling, Braja and Subrata have managed to prepare metoprolol tartarate tablets comprising the drug and chitosan through DC [95]. The contribution of chitosan to the compression properties was compared to that of tablets made with Eudragit RL-100, ethyl cellulose, hydroxyethyl cellulose (HEC), and hydroxypropylmethyl cellulose (HPMC) K-100. When *P_Y_* and “*a*” of the Heckel and Kawakita equations were used to estimate the compressibility parameters, the plasticity appeared to follow the order chitosan > Eudragit RL 100 > ethyl cellulose > HPMC K100 > HEC. However, estimation of powder properties upon compression was carried out using a hydraulic tablet press with one minute dwelling time. Ching *et al.* emphasized that the tabletability and compressibility of plastic and brittle pharmaceutical powders are speed-dependent [96].

The plasto-elasticity behavior of chitin was quantified (Figure 8) along with Avicel^®^ PH 102 and Cellactose^®^ from the force distance curve using Antikainen and Yliruusi’s method [94]. Avicel^®^ PH 102 and chitin mainly undergo plastic deformation during compression [94,97]. Higher plasticity, in general, would lead to more contact points for interparticulate bonding [98]. This justifies the highest tensile strength displayed by Avicel^®^ PH 102 tablets, followed by chitin and Cellactose^®^ (Figure 8). For Cellactose^®^, despite its low cellulose content, the binding between lactose particles is mediated by cellulose [97,99].

On the other hand, elastic deformation causes a profound decrease in the tensile strength of tablets. This is due to disruption of interparticulate bonds when the compaction pressure is released. As a material exhibiting high plastic deformation, Avicel^®^ PH 102 has a minimal tendency to encounter a reversible deformation behavior. Of the values for excipients’ elasticity factor in Figure 8, chitin’s elasticity is the highest, followed by Cellactose^®^ and Avicel^®^ PH 102. This higher elasticity therefore contributes to the lowest tensile strength observed with chitin tablets.

### 2.5. Factors Contributing to the Powder Compressibility Properties of Chitin/Chitosan

#### 2.5.1. Moisture Content

It is generally accepted that moisture content has the largest impact on powder compressibility [70,71]. Increasing the moisture content decreases the granule porosity and fragmentation propensity and hence increases granule strength [100]. As seen in Figure 8, the values for the plasticity factor (PF) initially increased for chitin, Cellactose^®^, and Avicel^®^ PH 102, reaching the maximum at 1.5%–2.5%, 3%–5%, and 7%–10% *w*/*w* moisture, respectively. The highest values of PF (4.4%) were obtained for Avicel^®^ PH 102, whereupon the PF value was clearly decreased as the moisture content was increased up to 14% (at 95% RH) as a consequence of free water effect.

#### 2.5.2. Degree of Deacetylation

At present, there is no solid data on the effect of chitosan’s degree of deacetylation on powder behavior upon compression. However, Picker-Freyer *et al.* noted that according to the DDA no order in powder compressibility could be set up [86].

#### 2.5.3. Molecular Weight

Heckel and Kawakita analyses were investigated by Rashid *et al.* and Qandil *et al.* on different MW chitosan powders of the same DDA [6,16]. With regard to the Kawakita parameter *a*, Table 3 shows the highest porosity is obtained for the highest MW chitosan, whereas the lowest porosity is obtained for the lowest MW chitosan. On the other hand, the amount of plastic deformation decreases (higher *1*/*b* values) for the lower MW chitosan samples. The foregoing was further verified by the yield pressure parameter (*P_Y_*) of Heckel analysis, which revealed that the higher the chitosan MW the higher its plastic deformation upon compression. This explains the increase in tensile strength for lower MW chitosan samples (presented in Figure 6), especially at low compression loads (<300 kg). It has to be noted that the extent of die filling and particle rearrangement before compression (*A*) for all samples is nearly the same (Table 3), probably due to similar particle size distribution of each of the chitosan samples.

**Table 3:** The Kawakita and Heckel parameters of chitin and chitosan of different molecular weights. Data were obtained from Rashid *et al.* [6].

Material/MW (kDa)	Kawakita Parameter					
	*a*	*ab*	*b*	*1*/*b*	*P_Y_*	*A*
Chitin	0.818	0.077	0.094	10.57	-	-
Chitosan/100	0.75	0.092	0.12	8.15	72.5	0.42
Chitosan/30	0.54	0.066	0.12	8.14	98.0	0.46
Chitosan/18	0.63	0.084	0.13	25.55	106.4	0.47
Chitosan/8	0.52	0.024	0.046	21.55	153.9	0.60

Kawakita *a*, *ab* and 1/b parameters represent porosity, extent of fragmentation, and plastic deformation, respectively. Heckel *P_Y_* and A parameters represent yield pressure and particle rearrangement, respectively. MW represents molecular weight.

In a related study presented by Katharina *et al.* wherein they investigated the slope of the Heckel function for different MW chitosans, chitosan with a MW of 87.2 kDa behaves in a manner similar to Avicel^®^ PH 102, and the others with 173.3 and 210.5 kDa showed a lower Heckel slope and thus higher resistance against deformation [86]. They suggested that the higher Heckel slope is the result of the lower MW of the chitosan. These results contradict the findings by Rashid *et al.* on chitosan plasticity [6]. It can be postulated that such inconsistency is related to the differences in the physical characteristics of the chitosans used. For example, the 173.3, 210.5, and 87.2 kDa chitosans differ in DDA, bulk density, and particle size. Each of these differences affects the extent of packing and deformation extents upon compression. In addition, the determination of the Heckel slopes includes plastic and elastic deformation, and therefore the contribution of the undetermined elastic recovery might have contributed to the Heckel slope.

A clearer judgment on the plasticity-MW relationship of chitosan can be drawn from the results presented by Chen and Hwa, who explored the effect of MW of chitosans with the same DDA on the elasticity (elongation at break) and tensile strength of chitosan films [101]. These films would have lost the contributions of particle size and size distribution to these parameters. Both parameters of the membranes prepared from high MW chitosan were higher than those of low MW chitosan.

### 2.6. Compressibility Changes upon Formulation and/or Modification of Chitosan

There are numerous applications of chitosan described in the literature, especially when it was used in solid dosage form. However, few studies have investigated the powder compression properties and tabletability of chitosan in relation to its physical and/or chemical modification or even to its simple role as an additive.

#### 2.6.1. Physical Mixing

A combination of chitosan with other polymers has been used either to provide a desired function for controlled or immediate release products or to improve the compressibility of chitosan. For example, Knapczyk showed that chitosan of 66% DDA, when used as a filler or binder in DC processing, produced tablets that suffer from low mechanical resistance but did not affect mass flow nor undermine rapid tablet disintegration [102].

In the preparation of sustained release DC tablets of ciprofloxacin HCl with a 1:1 ratio of chitosan and gum kondagogu, the powder’s and tablets’ physical properties were evaluated [103]. The powder mixtures comprising the drug, the polymeric matrix, and starch have recorded bulk densities of 0.43–0.48 g/mL. In other words, the drug (bulk density of 0.2 g/mL) has been formulated into a compressible powder by physical mixing, reaching double its own bulk density. Furthermore, a CI of 3.4%–6.16% was achieved. It is clear that the contribution of the bulk densities and the improvement in the flow of the preparations are more likely attributed to starch (~0.55 g/mL), which is present at a high level, and to gum kondagogu (CI of 16% and bulk density of 0.704 g/mL) [103].

A similar approach involving chitosan physically mixed with other hydrophilic polymers was utilized to produce sustained release DC terbutaline sulfate tablets [104], employing chitosan and xanthan gum mixed at a 1:1 mass ratio with sodium bicarbonate as a release-modifying agent. Although the properties of the physically mixed powders were not recorded, results of drug content, friability, weight variation, and tablet hardness from the three batches’ reproducibility data indicated batch-to-batch reproducibility, and no significant differences were noticed. Accordingly, the requisite powder’s flow properties are achieved with such a polymeric combination. Such improvement in flow is anticipated by the high bulk density of sodium bicarbonate (~1.0 g/mL), which should ultimately improve the extent of die filling.

**Figure 9:**
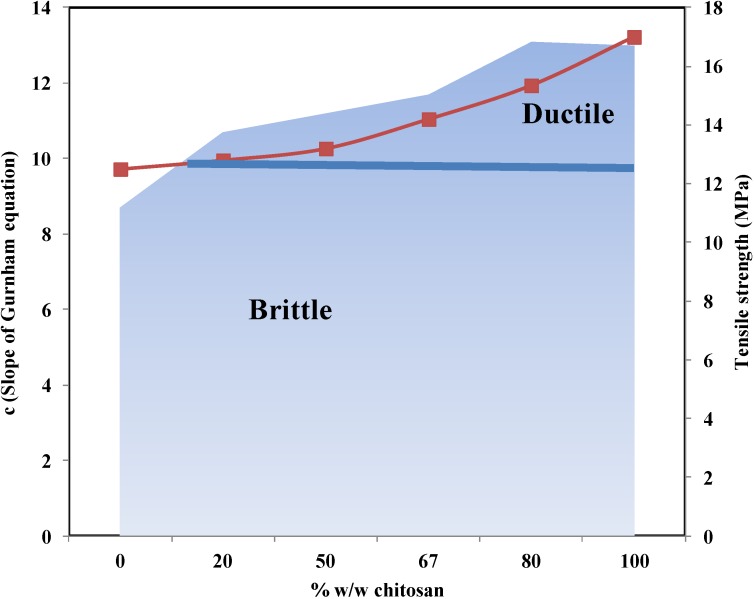
The effect of chitosan content on the plasticity of a chitosan–xanthan mixture. Data were obtained from Eftaiha *et al.* [107].

The effect of chitosan on powder compressibility when present with other polymeric excipients (e.g., xanthan gum) was described by Eftaiha *et al.* [105]. They correlated the compression to the relative bulk and tapped density of chitosan and xanthan gum, from which the porosity was determined according to Equation (9):

(9) ε=1−ρ

where ε is porosity and ρ is the relative density. Accordingly, the porosity of the bulk and the tapped powder for chitosan is 0.879 and 0.842, while for xanthan gum it is 0.571 and 0.510, respectively. Based on the porosity values, chitosan is more porous than xanthan gum, giving rise to a higher extent of volume reduction upon compression for chitosan than for xanthan gum. The foregoing was verified using force-displacement curves, whereby chitosan showed a high displacement when compared to xanthan upon compression. Furthermore, the Gurnham equation (Equation (6)) was used to investigate different mixtures of chitosan and xanthan gum. Since ductile and brittle materials demonstrate large or little amounts of plastic deformation before fracture, respectively [106], according to Gurnham’s model and Zhao’s classification, xanthan gum is a brittle material, whereas chitosan is a ductile material (Figure 9). Furthermore, plasticity increases as the mass fraction of chitosan is increased and the 1:1 chitosan:xanthan gum mass ratio represents a reasonably ductile combination of sufficient tensile strength. The foregoing ratio was estimated to present a percolation threshold that generally takes place between plastic and elastic materials at specific concentrations.

#### 2.6.2. Spray-Drying

Due to the fact that spray-drying offers a means of obtaining powders of predetermined particle size and shape [108], most spray-drying processing of chitosan was carried out mainly to improve powder flow. Thus powder compressibility and in most instances powder compactibility are improved. The use of spray-dried chitosan as an excipient for directly compressible tablet formulations has been reported earlier [109]. The suitability of chitosan in the formulation of sustained release dosage forms of a few drugs has been disclosed [110,111,112]. Although chitosan is insoluble in water, its solubility in weakly acidic media makes it available for processing by spray-drying. On the other hand, once dissolved, it forms protonated chitosan amines. The acquired positive charges can result in precipitation of complexes or insoluble salt forms of chitosan upon addition of negatively charged molecules during processing [113].

Rege *et al.* spray-dried chitosans of different MW and compared these products with tray-dried chitosans of equivalent MW [109]. The bulk density of spray-dried chitosan, in general, was higher than tray-dried chitosan. The foregoing is correlated to the powder packing characteristics, where the small, spherical, spray-dried particles are expressed to closely pack together and thus lower the volume-to-mass ratio; consequently, a higher bulk density is obtained. On the contrary, the large, irregular, tray-dried particles showed poor packing characteristics with a lower bulk density. With regard to compressibility, spray-dried chitosans, in general, exhibited a lower percentage of compressibility in contrast to tray-dried chitosans. This difference corresponds to the improved flow property of the spray-dried material [114]. Such behavior is attributed to the fact that spray-dried particles, in general, are hollow spheres, with lower particle density than the relatively solid tray-dried particles. With regard to the effect of MW on the properties of spray-dried chitosan, although the tablets’ tensile strength was not measured, Rege *et al.* highlighted that, upon preparation, tray-drying invariably resulted in hard tenacious masses that needed vigorous milling, whereas spray-dried granules did not require additional milling [109,115]. Nevertheless, in a separate study, Rege *et al.* managed to test the tensile strength of tetracycline-chitosan tablets prepared by spray- and tray-drying techniques [109]. They concluded that the tensile strength of tablets containing spray-dried chitosan was generally higher than in those containing tray-dried chitosan at each MW. Furthermore, the tensile strength of low MW chitosan tablets (2–16.6 kDa) was higher than that of tablets containing chitosans of MW 280–519 kDa.

Similar conclusions regarding powder flow and compressibility properties were drawn when a naproxen sodium–chitosan complex was spray- and tray-dried [116]. The significant result in this work is the disappearance of the crystalline structure of the naproxen–chitosan complex upon spray-drying, as naproxen and chitosan have highly and partially crystalline structures, respectively. Such crystallographic modification is further confirmed upon spray-drying of theophylline–chitosan complexes [110]. Theoretically, as the percent relative crystallinity increases, the crushing strength of tablets also increases [117]. Crystalline regions have a highly ordered arrangement of molecules in their structure. Upon compression of crystalline materials, the crystalline regions are forced to become closely packed. The tighter molecular arrangement, in combination with the high plastic deforming ability of e.g., microcrystalline cellulose and starch, facilitates the formation of hydrogen bonding upon compression, resulting in the formation of strong compacts [6,117]. The aforementioned fact contradicts with the reported high tensile strength of chitosan complexes, which became amorphous after being subjected to spray-drying. Upon compression of spray-dried chitosan, more particle–particle contact occurs, either by plastic or brittle deformation, as the next examples elucidate.

**Figure 10:**
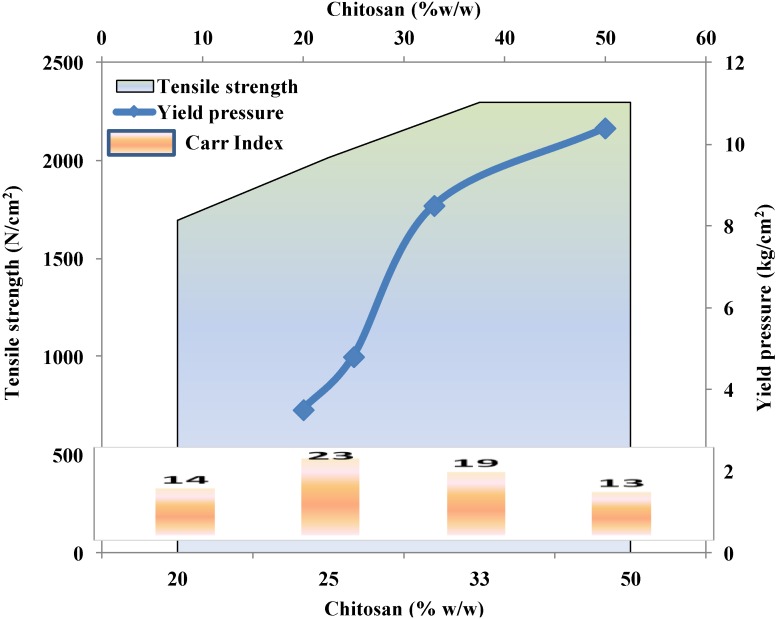
Effect of chitosan content on powder and tablet characteristics of spray-dried mixtures of chitosan and hydrolyzed gelatin. Data were obtained from Kokil *et al.* [118].

Figure 10 presents the powder and tablet characteristics of spray-dried mixtures of chitosan and hydrolyzed gelatin. Initially, at all concentrations of chitosan, the powders show better flow characteristics, with higher tensile strength, than hydrolyzed gelatin itself. Increasing the content of chitosan favors an increase in the yield pressure, which implies that plasticity decreases with an increase in the concentration of chitosan. However, a decrease in plasticity results in lower contact surface area and therefore lower tensile strength of the compacts. This contravenes the trend toward an increase in tensile strength as the chitosan content is increased (Figure 10). Suruchi *et al.* suggest that this could be attributed to the formation of low-strength granules that fracture readily, resulting in a significant increase in particle–particle contact surface area, which leads to enhanced bonding between adjacent particles [118].

#### 2.6.3. Co-Precipitation of Silicon Derivatives on Chitin/Chitosan

In the search for new multifunctional excipients with strong binding, good flow, and fast disintegration characteristics, there has been a focus on silicon derivatives. Initially, silicon dioxide was co-precipitated onto chitosan and chitin at a 1:1 mass ratio [10,50,51,119]. In addition to enhanced binding and disintegration properties, the compressibility improved in comparison to their initial properties. For example, the 0.27 g/mL bulk densities of chitin increased to 0.45 g/mL for a chitin–silicon dioxide co-precipitate and the powder compressibility is 7.3% for a chitosan–silicon dioxide co-precipitate (Table 4). Consequently, the powder flow has earned an “Excellent” rank under the CI criteria. Furthermore, as evidenced by the *D_A_* and *D_B_* values, the chitin–silica co-precipitate undergoes a high extent of packing and rearrangement in comparison to Avicel^®^ 200 (microcrystalline cellulose of particle size 180 µm). With regard to powder deformation under compression, both Avicel^®^ 200 and the co-processed excipients undergo plastic deformation to a similar extent when their yield pressure (*P_Y_*) values are considered. However, a faster onset of plastic deformation (*1/b*) takes place by individual (unprocessed) excipients due to their higher compressibility (*a*) and high particle rearrangement (*ab*).

The change in powder compressibility due to formation of the co-precipitates can be attributed to the dramatic change in chitin or chitosan structures, from thin flat surfaces, some with irregular folded edges, to three-dimensional compacts of chitin–silica. The aforementioned was evidenced by an almost 40% reduction in the measured specific surface areas for the co-precipitates when compared to their native excipients. However, the partially crystalline nature of chitin was still intact. Ultimately, co-processing of silicon dioxide/silicate onto chitin or chitosan brings about freely flowing and denser packing particles without significantly affecting the plasticity of the native excipient or its crystalline structure, unlike what is observed with spray-drying.

**Table 4:** The powder properties of chitin and chitosan mixtures with silicate excipients, in comparison with co-processed microcrystalline cellulose (Avicel^®^ PH 200). Data were obtained from El-Barghouthi *et al.* [119] and Rashid *et al.* [50,51].

Mixture	BD	TD	% Comp.	*Heckel Parameters*			*Kawakita Parameters*			
				*P_Y_*	*D_A_*	*D_B_*	*a*	*b*	*1/b*	*ab*
Chitosan–silica (50% chitosan)	0.38	0.41	7.32	–	–	–	–	–	–	–
Chitin–silica (50% chitin)	0.45	0.5	10	98	0.165	0.588	–	–	–	–
Chitin–Mg silicate (68% chitin)	–	–	–	–	–	–	0.75	–	17.37	0.043
Chitin	0.27	0.39	30.77	–	–	–	0.82	1.67	0.6	0.077
Avicel^®^ 200	–	–	–	81.3	0.09	0.611	–	–	–	–

Kawakita *a*, *ab*, and 1/b parameters represent porosity, extent of fragmentation, and plastic deformation, respectively. Heckel *P_Y_*, *D_A_*, and *D_B_* parameters represent yield pressure, the total degree of densification, and the phase rearrangement of particles during the initial stages of compression, respectively. BD and TD represent the bulk and tapped densities, respectively.

The interaction of co-processed chitin–metal silicate excipients with model drugs was investigated in aqueous slurry and in the solid state [120]. When cefotaxime sodium was chosen as a model drug that exhibits acid or base catalysis, chitin–aluminum silicate showed minimum drug instability in the solid state, close to where the maximum drug stability in the slurry was observed. This was attributed to the catalytic properties of chitin–aluminum silicate. Generally, catalytic activities are associated with oxidation/reduction and acid/base reactions of the metal cation on the silica surface. Such activity depends on the polarizing power of the metal, which is a function of the effective nuclear charge and size of the cation. On the other hand, the slurry method did not efficiently predict the solid-state surface acidity and stability of cefotaxime sodium. Moreover, the solid-state chemical stability might be influenced by factors other than the solid-state acidity. The stability of cefotaxime sodium was achieved with chitin–magnesium silicate when the surface pH was almost neutral.

#### 2.6.4. Co-Processing of Chitin/Chitosan by Compaction

Roll compaction is a continuous dry granulation process that is widely employed in the pharmaceutical industry to manufacture free flowing agglomerates. In this technique, compacted ribbons or flakes are produced and then milled to form granules of desired size. Such mechanical densification results in an increase in the bulk density, thus producing high-quality tablets with high dose uniformity and low weight variation [3].

Compaction of chitosan was carried out for the purpose of characterizing the compaction properties of chitin samples of different MW (listed previously in Table 3) [121]. With respect to granules’ deformation mode, compacted chitin undergoes plastic deformation to a greater extent (lower *P_Y_*) than does native chitin or even any or the co-precipitated excipients listed in Table 4. Such high plasticity was confirmed when chitin was compacted with mannitol at a 20:80 (mannitol:chitin) mass ratio. The plasticity was identified from the low Kawakita parameter (*1*/*b*) of the compacted mixture, 7.8, compared to that of the individual components (10.6 for chitin and 10.3 for mannitol). Such high plasticity is responsible for the high tensile strength of tablets made from the co-processed excipient. Compressibility (*a*) of the compacted mixture is nearly the same for chitin and is relatively low for mannitol (*a* = 0.576). Therefore, co-processing of chitin with mannitol provides no substantial increase in the compressibility of chitin. At 20% level, mannitol occupies only a limited area of the large surface pores of chitin. These pores are responsible for the decrease in the volume of chitin powder upon compression [122].

## 3. Conclusions

Pharmaceutical applications of chitin and chitosan and their derivatives as effective excipients can be aligned for DC processing. The diversity of physico-chemical properties of semi-crystalline nature, DDA, and MW enhance beneficial use as such and as a co-processed excipient in pharmaceutical preparations. Moreover, their high surface area, porous structure, and plastic deforming nature enhance necessary particle bonding and tabletability in the DC mode with low sensitivity upon lubrication. Optimal use as a single multifunctional excipient can be established when chitin and chitosan are co-processed with other excipients.
